# General Remarks on Some of the Pathological Conditions of the Naso-Pharynx

**Published:** 1890-10

**Authors:** A. B. Patterson

**Affiliations:** Late Assistant to the Royal London Ophthalmic Hospital and Golden Square Throat Hospital, London, England


### GENERAL REMARKS ON SOME OF THE PATHOLOGICAL CONDITIONS OF THE NASO-PHARYNX.

BY A. B. PATTERSON, M. D.,

Late Assistant to the Royal London Ophthalmic Hospital and Golden Square Throat Hospital, London, England.

Naso-pharyngeal troubles and the complications they lead to, are, I am sure, of a more frequent occurrence than is generally recognized. Their prevalence in some portions of America—no doubt due to the peculiarities of our climate—in some way conducive to the catarrhal condition, the dusty, dry and heated atmosphere of our summers and the sudden changes peculiar to our winters, acting upon constitutions already debilitated from the effects of malaria. Naso-pharyngeal catarrh is a disease much dreaded by some of our people, owing to the popular belief that there is a tendency of this disease to pass into consumption. There are some grounds for these fears, as an inflamed and abraded mucous surface of the nose and pharynx are in a condition favoring the germination and introduction of tubercle-bacilli into the lymphatics. These germs, aeronautic on certain occasions, are taken into the air passages on motes and particles of dust. I fear the chronic diseases peculiar to the nose and throat are not receiving the consideration they deserve from the general practitioner. The pathology of this region and the reflex neuroses opening therefrom, are comparatively a new field of pathological research which would yield an abundant harvest if diligently cultivated. It may not be amiss to briefly survey the minute anatomy of the mucous surfaces that line the nose and pharynx. The former is lined by a vascular membrane, covered by epithelium cells of the bipolar or spindle-shaped variety and long columnar epithelium cells, forked, branching, tail-like, and are found next to the free surface, being projected in the neighborhood of the corium; they support the olfactory cells and are supposed to be connected with the olfactory fibers; the difference between these and the bipolar cells is not very marked. The latter cells consist, as the name implies, of two processes, cylindric and straight, and are found on the surface. Among these cells are projected the nerve filaments, glands and follicles, and is the ground upon which fermentative agencies implant themselves, and frequently produce damage irreparable.

The pharynx in its anatomical construction varies from that of the nose, being surrounded by the constrictor muscles, over which is the mucous membrane, covered by epithelium cells of ciliated type. In the lower region the epithelium is of the stratified type, not differing from that found in the buccal cavity; the pharynx is freely supplied with mucous glands and follicles, and in the upper and back part lymphoid tissue is found, frequently assuming unusual proportions, giving rise to the so-called pharyngeal tonsil. What are facts and truisms to some observers are rejected as fallacies by others. Such discrepancies might be accounted for in many cases in a deficiency in miscropical anatomy and morbid pathology. A knowledge of these branches will enable the observer to comprehend many phenomena which heretofore were perplexing and confusing. Wokes' theory in regard to the etiology of catarrh is quite ingenious; that there are other factors to be considered in discussing the etiology of catarrh or inflammation of mucous surfaces cannot be doubted, after a study of the modern theory of disease.

The mechanism of taking cold, as elaborted by Wokes, cannot be accepted as the cause of the pathological changes as seen in naso-pharyngeal catarrh. The mobid impressions conveyed to the epithelium cells, by way of the sympathetic chain of nerves, are impressions of a debilitating character lowering the resistance of the cells to extraneous matter, which, if fermentative in nature, will set up phenomena spoken of as inflammation. The sympathetic system of nerves has under its control the nutrition of the tissues. I cannot accede to the theory that a morbid condition of the nerves, producing an arrest of nutrition, can result in the pathological condition known as inflammation. The resistance of the cells having been lowered, they offer to germs a home and nutrition that favor their development.

Modern methods of research in histology, pathology and bacteriology, the latter with its laws of fermentation and the resulting alkaloids, many of which are of a poisonous character, explain many phenomena that heretofore could only be speculated upon. If pathological changes in the nasal cavities of an obstructive nature should take place, necessitating breathing through the mouth, very soon the pernicious habit will set up the most distressing symptoms, which frequently lead to serious consequences.

The nasal cavities filter and heighten the temperature of the air, thus preparing it for the air cells and delicate blood currents. That laryngitis, bronchitis, asthma, cough and neuralgia frequently have their origin in the naso-pharyngeal cavity cannot be doubted. It has long been the opinion of many observers, that inflammation has a tendency to pass from above downwards, and not from below upwards. Every practitioner of experience knows how unyielding some of these troubles are to therapeutical agencies. Success only attends surgical interference; hypertrophic tissues are to be reduced, polyps and lymphoid papillomata are to be removed, the mucous membrane must be kept clear of tenacious and fermentative secretion; this is accomplished by systematically removing the mucus by cotton brushes, and a thorough cleaning with antiseptic sprays.

The successful management of diseases of the naso-pharyngeal cavity depends upon an early recognition and treatment, as the tendency of all inflammation is to destruction of tissue elements. It must not be presumed that after hypertrophy has lasted for some time and atrophy has set in, that we can by any possible means restore the tissues to their once normal condition, though much can be done towards making the patient comfortable; these facts should be urged upon the patient by his attendant.

The following cases will bear me out in much that I have had to say, and may be of interest to some of your readers. A young man came under my care, suffering with an inflammation of the conjunctiva and cornea of the right eye, of several years' duration. He stated his physician frequently succeeded in curing the inflammation, but it would commence again on leaving off the remedies. An examination revealed the fact that both nostrils were the seat of hypertrophic tissue and polyps, the nostril corresponding to the eye affected was quite occluded. After a thorough renovation of the nasal cavity, the eye trouble yielded to treatment, which has shown no disposition to return. These cases are not unusual, infection being conveyed to the eye from the nose by way of the lachrymal canal.

I have recently discharged a young man who had a cough attended with profuse expectoration. His family were anxious about him, as he was losing flesh and spirits. It was thought he was passing into consumption. Tonics and cough mixtures had been tried without relief. I discovered that the expectorated sputæ was from the pharynx, attended with great thickening of mucous membrane, with a co-existing hypertrophic catarrh of the nose. The catarrh was due to the dropping of the secretion from the pharynx into the larynx setting up a laryngitis. After a thorough cleaning of the naso-pharynx, the profuse secretion yielded to applications made directly to the mucous surface of the pharynx. The laryngitis subsided after a few astringent applications, and a course of medicated inhalations; these complications having been removed, the boy has regained his flesh, and is now a jolly, happy fellow.

Acute and chronic inflammations of the middle ear, suppuration and non suppuration have their origin in the pharynx; relief of the deafness and cure of the catarrhal condition of the middle ear depend upon early and vigorous treatment of the pharynx. All efforts to relieve by injections, simple and medicated into the ear invariably meet with failure, and in many instances do harm. In concluding these rather unconnected and rambling remarks, I wish to direct the attention of your readers to one other condition of the pharynx, which is by no means uncommon. I refer to the enlarged tonsils of the honey-comb variety. They become the seat of abscesses, to the great discomfort of the patient. The honey-comb recesses become the hot-bed for fermentative matter, which is constantly being taken into the stomach by the act of deglutition, and on to the blood current, vitiating the nutrition and poisoning the tissues; further the pernicious effect upon the organ of hearing, and the perpetuating a chronic pharyngitis. The treatment is complete ablation.

*27 Old Capitol Building.*

